# Bilateral Asymmetric Traumatic Dislocation of the Hip: Case Report

**DOI:** 10.1055/s-0044-1779325

**Published:** 2024-04-22

**Authors:** Mauricio Hoffmann Sanagiotto, Ricardo Issler Unfried, Tiango Aguiar Ribeiro

**Affiliations:** 1 Departamento de Ortopedia e Traumatologia, Hospital Universitário de Santa Maria, Universidade Federal de Santa Maria, Santa Maria, RS, Brasil

**Keywords:** accidents traffic, acetabum, fractures, bone, hip dislocation

## Abstract

Traumatic hip dislocations are serious traumas, the result of high-energy mechanisms, constituting one of the orthopedic emergencies. The epidemiology of asymmetric bilateral hip dislocation is an extremely rare orthopedic condition. Prompt diagnosis and urgent reduction are essential due to the high risk of future complications. The objective of this report is to describe the case of an 81-year-old man, a victim of a car accident who presented with traumatic bilateral asymmetric dislocation of the hip, aiming to increase the limited data on the topic, through discussion of the mechanism, management, follow-up and epidemiology.

## Introduction

Bilateral hip dislocations account for 1% of all hip dislocations. 1 Bilateral asymmetric hip dislocation is even rarer, constituting only 0.01% of all dislocations. Asymmetrical hip dislocations are uncommon because the force vectors required are exactly opposite at each hip. The mechanism of trauma of these injuries is generally related to a high kinematic, associated with acetabular fractures. 2 They are more common in young male patients. 3 Compared to the last century, the incidence of asymmetric hip dislocations has increased considerably as a result of traffic accidents. 4 In this report, we present a rare case of bilateral asymmetric hip dislocation associated with acetabular fracture.

## Case Report

This case report was approved by the research ethics committee (CAAE: 70203223.1.0000.5346- opinion 6,114,087). Male patient, 81 years old, previously healthy, retired farmer, victim of a car rollover in January 2022, without wearing a seat belt. After the trauma, he was taken and admitted to the emergency room at the Santa Maria University Hospital.

**Fig. 1:**
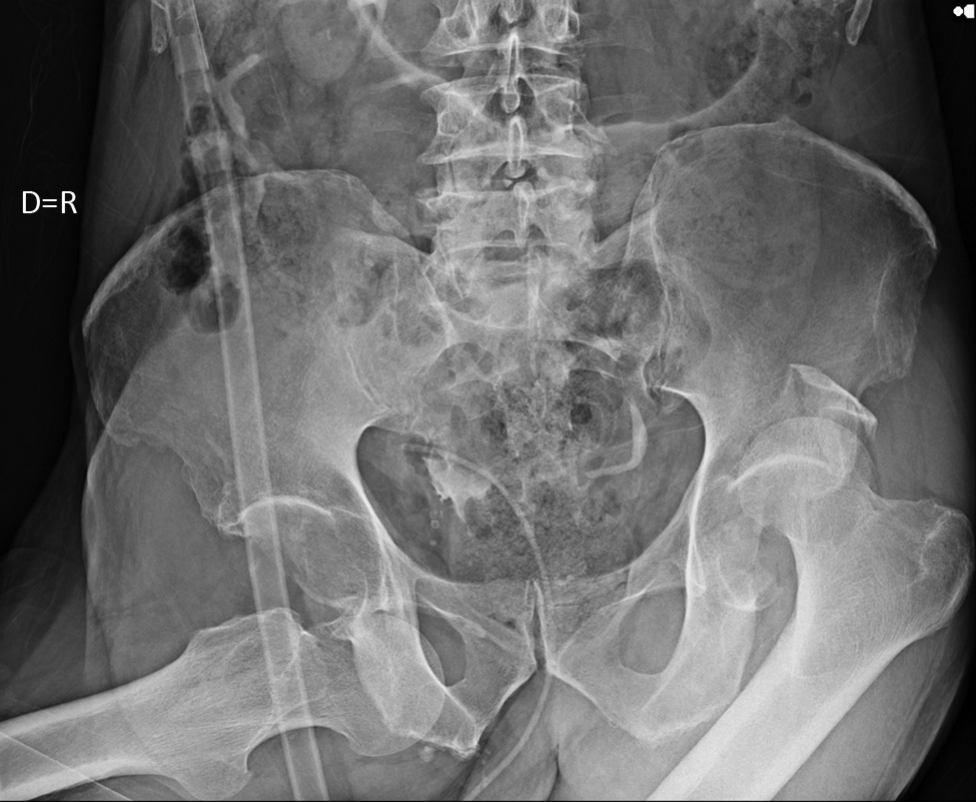
AP radiograph of the pelvis showing asymmetric bilateral dislocation of the hips.

At initial care, the right lower limb was in flexion, abduction and external rotation of the hip. The left lower limb was in flexion, adduction and internal rotation of the hip. Both lower limbs had preserved neurovascular conditions. A pelvic X-ray revealed asymmetric bilateral dislocation of the hip, anterior to the right and posterior to the left, the latter associated with acetabulum fracture ( Fig. 1 ). Fractures of the first to ninth costal arches on the right, pleural effusion, fracture of the sternal manubrium and fracture of the right transverse process of the first lumbar vertebra were also diagnosed.

**Fig. 2:**
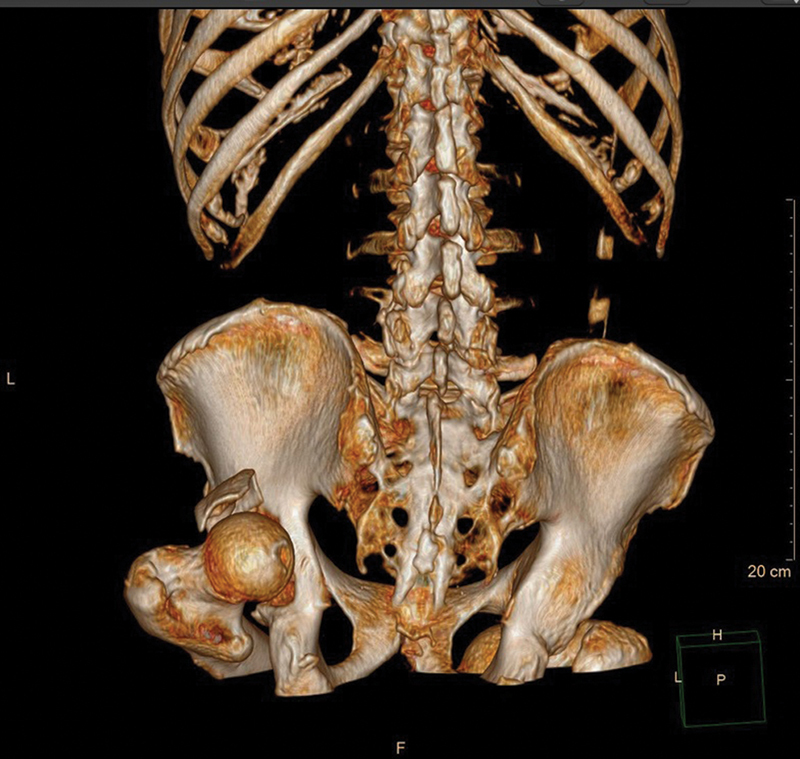
Computed tomography of the pelvis (3D reconstruction) showing the fracture of the posterior wall of the left acetabulum.

After performing a computed tomography of the pelvis, the dislocation of the left hip was classified as posterior, with an associated fracture of the acetabulum ( Fig. 2 ), presenting a large and single fragment of the posterior wall (Thompson and Epstein type II). In the right hip, the hip dislocation was classified as anterior and inferior, with no signs of acetabular fracture (Epstein type IIA).

**Fig. 3:**
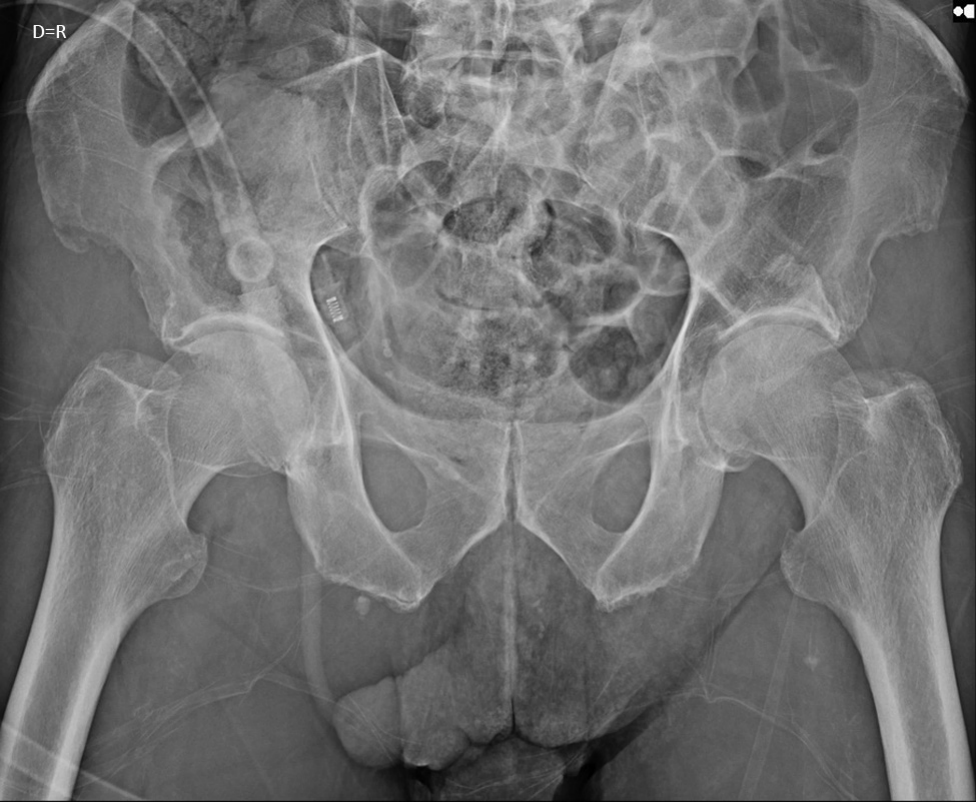
AP radiograph of the pelvis after closed reduction of asymmetric bilateral dislocation of the hips.

After initial clinical stabilization, in a stable hemodynamic condition, the patient was taken to the surgical suite and sedated by the anesthesiology team, where closed reduction maneuvers were performed for both hip dislocations. With the patient in the supine position, Allis maneuvers were performed on both hips, verified by radiographic control, which confirmed concentric and stable joints ( Figs. 3 e 4 ), without the need to use skeletal traction. No significant difficulties were encountered during the hip reduction maneuvers.

**Fig. 4:**
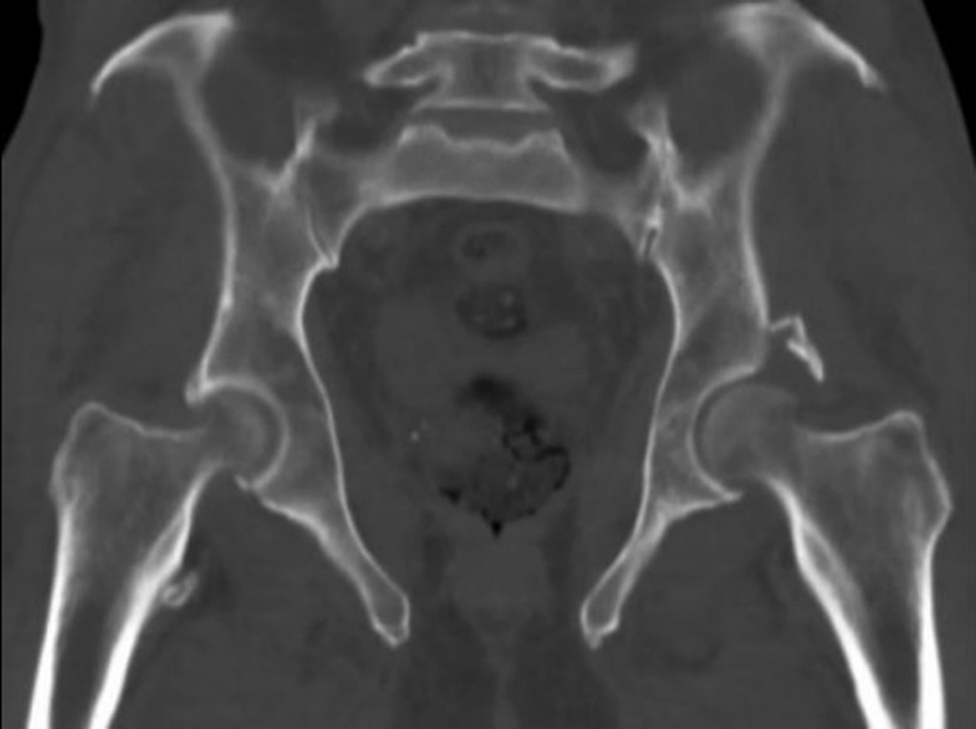
Computed tomography of the pelvis for surgical planning of fracture of the posterior wall of the left acetabulum.

**Fig. 5:**
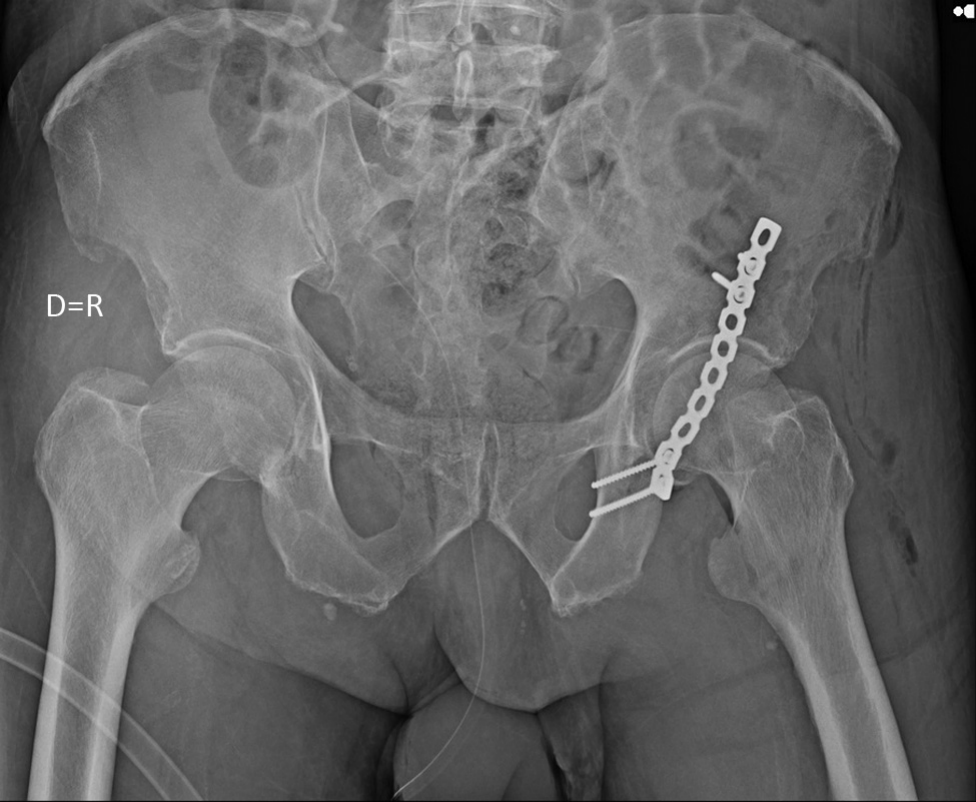
AP radiograph of the pelvis showing postoperative fracture of the posterior wall of the left acetabulum.

After 6 days of hospitalization, a new surgical intervention was performed to correct the left acetabular fracture. The access route used was posterior (Kocher-Langenbeck) and the implant used was a 3.5 mm reconstruction plate with 10 holes and 4 3.5 mm cortical screws ( Fig. 5 ). No complications occurred during or after the procedure. During the operation, the stability of both hips was tested again, which remained stable.

After 2 weeks of hospitalization, the patient was discharged from the hospital. Outpatient follow-up was not carried out due to death from COVID-19 2 weeks after hospital discharge.

## Discussion

Asymmetric bilateral hip dislocations often result from high-energy trauma, given that the anatomy of the hip is composed of resilient ligamentous structures. The male gender is the most prevalent (81%), and the average age is 32 years, usually associated with injuries in other body areas about 95% of the time. 3 5

Also known as "wind-swept deformity", asymmetric hip dislocations are rare due to the forces required to generate an anterior hip dislocation being exactly opposite to those needed to reproduce a posterior dislocation. 2 6 The mechanism of trauma in posterior dislocations results from an axial compressive force directed at the knee while the hip is in flexion and adduction. Conversely, in anterior hip dislocations, the force is applied with the limb in abduction and external rotation. 7

With asymmetric hip dislocations, there appears to be no indication to reduce the anterior or posterior hip first. Buckwalter et al. 3 state that it is possible that there is some type of endocrine effect after the reduction of one hip that allows more muscle relaxation and effectively an easier reduction of the second hip. In 88% of reported cases, closed reductions of both hips were performed, close to 92% of simple hip dislocations. Neither time was it necessary to open reduction of both hips. 3 As in symmetrical bilateral or unilateral dislocations, they are also associated with a better prognosis when the reduction is performed within 6 hours of the trauma. 2

Regarding outcomes, 61% of patients with asymmetric hip dislocations returned to normal strength, absence of pain and full range of motion (ROM) while 31% evolved with mild pain, small change in ROM or some non-damaging loss of strength. The remainder of patients developed impairment or changes in lifestyle due to long-term complications, generally found in patients with multiple injuries. 3

The significant majority of cases of asymmetric hip dislocations are not exposed. The first reported case of bone exposure was described in 2023 by Santoso et al. 8 Associated fractures were reported in 44% of patients, with acetabulum fracture accounting for 75% of these fractures. 2

Negligence in asymmetric hip dislocation is considered when the dislocation time is more than 1 week. 9 The treatment of neglected hip dislocations is complex, due to the formation of fibrous tissues in the joint. In these cases, open reduction or total hip arthroplasty achieved satisfactory results. The case with the longest period of neglect found in the literature was 7 months. 8

The first published case report of asymmetric hip dislocation occurred in 1845. 3 According to the literature, until 2001, only five cases of asymmetric bilateral hip dislocation were known. 10 In 2015, Buckwalter et al. 3 presented a comprehensive review and concluded that there were around 104 registered. In the period between 2015 and 2021, 17 more cases were identified, increasing to 121 cases in total. 3 6
