# Supplementary material for: Bilateral Asymmetric Traumatic Dislocation of the Hip: Case Report
Source: Rev Bras Ortop (Sao Paulo). 2024 Apr 22;59(Suppl 2):e247–50. doi: 10.1055/s-0044-1779325 (PMC11679615; doi:10.1055/s-0044-1779325)
Supplement: Related texts

# Research article: Luxação traumática bilateral assimétrica do quadril: Relato de caso

**Keywords:** acetábulo, acidentes de trânsito, fraturas ósseas, luxação do quadril

## Abstract

Luxações traumáticas do quadril são traumas graves, resultado de mecanismos de alta energia, constituindo-se uma das emergências ortopédicas. A epidemiologia da luxação bilateral assimétrica do quadril se apresenta como uma condição ortopédica raríssima. O diagnóstico breve e a redução urgente são essenciais, devido ao alto risco de complicações futuras. O objetivo deste relato é descrever o caso de um homem de 81 anos, vítima de acidente automobilístico que apresentou luxação traumática bilateral assimétrica do quadril, visando aumentar os dados limitados sobre o tema, através da discussão do mecanismo, manejo, seguimento e epidemiologia.

## Introdução

As luxações bilaterais do quadril representam 1% de todas as luxações do quadril. 1 A luxação bilateral assimétrica do quadril é ainda mais rara, constituindo apenas 0,01% de todas as luxações. Luxações assimétricas de quadril são incomuns devido aos vetores de força exigidos serem exatamente os opostos em cada quadril. O mecanismo de trauma destas lesões geralmente está relacionado à alta cinemática, associado a fraturas acetabulares. 2 São mais comuns em pacientes jovens do sexo masculino. 3 Em comparação ao século passado, a incidência de luxações assimétricas do quadril aumentou consideravelmente em decorrência dos acidentes de trânsito. 4 Neste relato, apresentamos um caso raro de luxação assimétrica bilateral de quadril associada a fratura acetabular.

## Relato de Caso

Este relato de caso foi aprovado pelo comitê de ética em pesquisa (CAAE: 70203223.1.0000.5346- parecer 6.114.087). Paciente do sexo masculino, 81 anos, previamente hígido, agricultor aposentado, vítima de capotamento de carro em janeiro de 2022, sem utilização de cinto de segurança. Após o trauma, foi conduzido e admitido no pronto socorro do Hospital Universitário de Santa Maria.

No atendimento inicial, o membro inferior direito se encontrava em flexão, abdução e rotação externa do quadril. Já o membro inferior esquerdo, estava em flexão, adução e rotação interna do quadril. Ambos os membros inferiores apresentavam condições neurovasculares preservadas. Na radiografia da pelve, evidenciou-se luxação bilateral assimétrica do quadril, anterior à direita e posterior à esquerda, a última associada a fratura de acetábulo ( Fig. 1 ). Também foram diagnosticados fratura do primeiro ao nono arcos costais à direita, derrame pleural, fratura de manúbrio esternal e fratura de processo transverso direito da primeira vértebra lombar.

**Fig. 1:** Radiografia em AP da bacia evidenciando luxação bilateral assimétrica dos quadris.

Após realização de tomografia computadorizada de bacia, classificou-se a luxação do quadril esquerdo como posterior, com fratura associada de acetábulo ( Fig. 2 ), apresentando fragmento grande e único da parede posterior (Thompson e Epstein tipo ll). No quadril direito, a luxação do quadril foi classificada como anterior e inferior, sem sinais de fratura acetabular (Epstein tipo llA).

**Fig. 2:** Tomografia computadorizada da bacia (reconstrução 3D) evidenciando a fratura da parede posterior do acetábulo esquerdo.

Depois da estabilização clínica inicial, em condição hemodinâmica estável, o paciente foi encaminhado ao bloco cirúrgico e sedado pela equipe da anestesiologia, onde foram realizadas manobras de reduções fechadas para ambas as luxações dos quadris. Com o paciente em posição de decúbito dorsal, foram realizadas manobras de Allis em ambos os quadris, verificadas por controle radiográfico que confirmaram articulações concêntricas e estáveis ( Figs. 3 e 4 ), sem necessidade de uso de tração esquelética. Não foram encontradas dificuldades significativas durante as manobras para as reduções dos quadris.

**Fig. 3:** Radiografia em AP da bacia após redução fechada da luxação bilateral assimétrica dos quadris.

**Fig. 4:** Tomografia computadorizada da bacia para planejamento cirúrgico da fratura da parede posterior do acetábulo esquerdo.

Depois de 6 dias de internação, foi realizada nova intervenção cirúrgica, para correção da fratura acetabular esquerda. A via de acesso utilizada foi posterior (Kocher-Langenbeck) e o implante utilizado foi uma placa de reconstrução de 3.5 mm de 10 furos e 4 parafusos corticais de 3.5 mm ( Fig. 5 ). Nenhuma intercorrência ocorreu durante ou após o procedimento. No transoperatório, foram testados novamente a estabilidade de ambos os quadris, os quais se mantiveram estáveis.

**Fig. 5:** Radiografia em AP da bacia mostrando pós-operatório da fratura da parede posterior de acetábulo esquerdo.

Após 2 semanas de internação, o paciente evoluiu com alta médica hospitalar. O acompanhamento ambulatorial não foi realizado devido a óbito por COVID-19 2 semanas após a alta hospitalar.

## Discussão

As luxações bilaterais assimétricas do quadril decorrem frequentemente de traumas de alta energia, dado que a anatomia do quadril é composta por estruturas ligamentares resistentes. O sexo masculino é o mais prevalente (81%) e a idade média é de 32 anos, associado geralmente a lesões em outras áreas corpóreas, cerca de 95% das vezes. 3 5

Conhecida também como “deformidade varrida pelo vento”, luxações assimétricas do quadril são raras em virtude das forças necessárias para gerar uma luxação anterior do quadril serem exatamente opostas àquelas necessárias para reproduzir uma luxação posterior. 2 6 O mecanismo do trauma em luxações posteriores resulta de uma força compressiva axial direcionada ao joelho, enquanto o quadril está em flexão e adução. De maneira oposta, em luxações anteriores do quadril, a força é aplicada com o membro em abdução e rotação externa. 7

Com luxações assimétricas do quadril, não parece haver indicação para reduzir primeiro o quadril anterior ou o posterior. Buckwalter et al. 3 refere que é possível que haja algum tipo de efeito endócrino após a redução de um quadril que permita mais relaxamento muscular e efetivamente uma redução mais fácil do segundo quadril. Em 88% dos casos relatados foram realizadas reduções fechadas de ambos os quadris, próximo aos 92% das luxações simples do quadril. Nenhuma das vezes foi necessário redução aberta de ambos os quadris. 3 Tal como em luxações bilaterais ou unilaterais simétricas, também estão associadas a melhor prognóstico quando a redução for realizada em até 6 horas do trauma. 2

Quanto aos desfechos, 61% dos pacientes com luxações assimétricas do quadril retornaram à força normal, ausência de dor e arco de movimento completo (ADM) enquanto 31% evoluíram com dor leve, pequena alteração na ADM ou alguma perda de força não prejudicial. O restante dos pacientes desenvolveram prejuízo ou mudança no estilo de vida devido às complicações a longo prazo, geralmente encontrados em pacientes com lesões múltiplas. 3

A maioria significativa dos casos de luxações assimétricas do quadril não são expostas. O primeiro caso relatado de exposição óssea foi descrito em 2023 por Santoso et al. 8 Foram relatadas fraturas associadas em 44% dos pacientes, sendo que a fratura do acetábulo constituiu 75% dessas fraturas. 2

A negligência na luxação assimétrica do quadril é considerada quando o tempo de luxação é superior a 1 semana. 9 O tratamento de luxações negligenciadas do quadril é complexo, devido a formação de tecidos fibrosos na articulação. Nesses casos, a redução aberta ou a artroplastia total do quadril obtiveram resultados satisfatórios. O caso com maior período de negligência encontrado na literatura foi de 7 meses. 8

O primeiro relato de caso publicado de luxação assimétrica do quadril ocorreu em 1845. 3 Conforme a literatura, até o ano de 2001, havia conhecimento de apenas cinco casos de luxação bilateral assimétrica do quadril. 10 Em 2015, Buckwalter et al. 3 apresentaram uma revisão abrangente e concluíram que havia cerca de 104 registrados. No período entre 2015 e 2021, mais 17 casos foram identificados, ampliando para 121 casos na totalidade. 3 6
